# Time Required for Nanopore Whole-Genome Sequencing of *Neisseria gonorrhoeae* for Identification of Phylogenetic Relationships

**DOI:** 10.1093/infdis/jiad170

**Published:** 2023-05-22

**Authors:** Laura T Phillips, Adam A Witney, Martina Furegato, Ken G Laing, Liqing Zhou, S Tariq Sadiq

**Affiliations:** Institute for Infection and Immunity, St George’s University of London, London, United Kingdom; Institute for Infection and Immunity, St George’s University of London, London, United Kingdom; Institute for Infection and Immunity, St George’s University of London, London, United Kingdom; Institute for Infection and Immunity, St George’s University of London, London, United Kingdom; Institute for Infection and Immunity, St George’s University of London, London, United Kingdom; Institute for Infection and Immunity, St George’s University of London, London, United Kingdom; Infection Clinical Academic Group, St George's University Hospitals NHS Trust, London, United Kingdom

**Keywords:** antimicrobial resistance, nanopore sequencing, *Neisseria gonorrhoeae*, surveillance, whole-genome sequencing

## Abstract

**Background:**

Antimicrobial resistance (AMR) in *Neisseria gonorrhoeae* is a global health challenge. Limitations to AMR surveillance reporting, alongside reduction in culture-based susceptibility testing, has resulted in a need for rapid diagnostics and strain detection. We investigated Nanopore sequencing time, and depth, to accurately identify closely related *N. gonorrhoeae* isolates, compared to Illumina sequencing.

**Methods:**

*N. gonorrhoeae* strains collected from a London sexual health clinic were cultured and sequenced with MiSeq and MinION sequencing platforms. Accuracy was determined by comparing variant calls at 68 nucleotide positions (37 resistance-associated markers). Accuracy at varying MinION sequencing depths was determined through retrospective time-stamped read analysis.

**Results:**

Of 22 MinION-MiSeq pairs reaching sufficient sequencing depth, agreement of variant call positions passing quality control criteria was 185/185 (100%; 95% confidence interval [CI], 98.0%–100.0%), 502/503 (99.8%; 95% CI, 98.9%–99.9%), and 564/565 (99.8%; 95% CI, 99.0%–100.0%) at 10x, 30x, and 40x MinION depth, respectively. Isolates identified as closely related by MiSeq, within one yearly evolutionary distance of ≤5 single nucleotide polymorphisms, were accurately identified via MinION.

**Conclusions:**

Nanopore sequencing shows utility as a rapid surveillance tool, identifying closely related *N. gonorrhoeae* strains, with just 10x sequencing depth, taking a median time of 29 minutes. This highlights its potential for tracking local transmission and AMR markers.

## Teaser

Early versions of nanopore technology demonstrate that very short sequencing times can accurately identify closely related *Neisseria gonorrhoeae* strains and key AMR markers, from cultured isolates, indicating promise as a real-time surveillance tool.

The spread of *Neisseria gonorrhoeae*, resistant to multiple antimicrobial classes, has created a major global health challenge [1–4]. Effective antimicrobial resistance (AMR) phenotypic surveillance programs provide early indication of AMR spread and inform treatment guidelines [3, 5]. Although for most antibiotic classes *N. gonorrhoeae* AMR prevalence exceeds the conventional 5% threshold for abandoning empirical use, variable numbers remain susceptible to these classes, depending on geographical region. In the 2019–2020 Gonococcal Resistance to Antimicrobials Surveillance Programme (GRASP) in England 55.7% *N. gonorrhoeae* strains were susceptible to ciprofloxacin [5] compared to >90% resistance in four Southeast Asian countries [3].

Directed *N. gonorrhoeae* treatment, enabled by culture and phenotypic susceptibility testing, has long turn-around times and has been largely replaced with nucleic acid amplification tests for diagnosis, impacting the ability of surveillance programs to disrupt AMR spread [6]. Molecular *N. gonorrhoeae* AMR genotypic markers correlate variably with phenotypic resistance due to the complexity of genetic-based resistance [7, 8]. However, some molecular markers are potentially useful in laboratory-based [9] or point-of-care tests [10–13]. For ciprofloxacin resistance, molecular AMR markers have remained relatively unchanged [14–16], associated mainly with the quinolone-resistance–determining region of the *gyrA* gene. For macrolides, penicillins, and tetracyclines, diverse mechanisms and continual evolution suggests molecular markers may be useful in combination with predictive modelling tools [15, 17].

Recent studies suggest whole-genome sequencing (WGS) from *N. gonorrhoeae* cultures has utility for molecular surveillance [18–21] and AMR prediction [22], highlighting collectively that the number of single-nucleotide polymorphisms (SNP) expected to contain all direct and indirect transmission pairs in samples collected a year apart may diverge by up to 14 SNP, and collected within the same day, between 0–9 SNP [18]. For this method to progress towards real-time surveillance, sequencing will be required directly from extracted clinical samples, which has been achieved using amplicon-based sequencing with high base accuracy for SNP detection [23], as well as in WGS studies for tracking *N. gonorrhoeae* AMR in high-risk sexual networks [19, 24]. Furthermore, real-time surveillance, which could support partner notification or contact tracing, might be deployable near clinical services using sequencing platforms that provide the flexibility to sequence to a requisite depth [25]. Oxford Nanopore Technologies’ MinION device has potential to be deployed near-clinic and be reuseable, thus effective in achieving these aims. Recent iterations of the technology have improved sequencing accuracy [26], and previous work demonstrated urinary and respiratory tract infection diagnosis and AMR detection from clinical samples within a four hour time frame [27, 28]. Various approaches to measuring *N. gonorrhoeae* AMR detection accuracy and speed have also been published, including an evaluating of the use of MinION Oxford Nanopore Technology sequences combined with Illumina sequences, to create de novo assemblies for accurate AMR determinant identification [29], and using a “genomic neighbor typing” approach using k-mers to rapidly predict phenotype based on matching to databases of reference sequences [30]. Recently, following 20 hours of sequencing clinical *Staphylococcus aureus* isolates on one flow-cell, accurate sequence type was obtained within 20 minutes, suggesting near-clinic applications are achievable if accuracy can be maintained [31].

We assessed the minimum MinION sequencing run time required for adequate sequence acquisition to accurately detect AMR variants and identify phylogenetically related *N. gonorrhoeae* strains, cultured from isolates collected from patients attending the same clinic within a three month period, as a first step towards an integrated rapid AMR surveillance tool.

## METHODS

### *N. gonorrhoeae* Isolates

*N. gonorrhoeae* isolates (n = 58) consecutively collected between July and September 2013 from a clinic in a London NHS Trust, as part of 2013 GRASP (UK Health Security Agency, formerly known as Public Health England), were retrieved from frozen growth in glycerol stocks at the Sexually Transmitted Bacteria Reference Unit and sent back to the clinic. All cultures had undergone phenotyping for antimicrobial susceptibility as part of GRASP, using European Committee on Antimicrobial Susceptibility Testing (EUCAST) breakpoints for minimum inhibitory concentration (MIC) [32].

### MiSeq Library Preparation and Sequencing

All 58 isolates were inoculated onto CBA (Oxoid) plates and incubated for 24–48 hours in 5% carbon dioxide at 35°C. A 10-µL loop of bacterial growth was used for genomic DNA extraction using QIAmp DNA Mini Kit (QIAGEN) according to manufacturer's instructions. Sequence libraries were prepared using Nextera XT library preparation kit (Illumina) according to manufacturer's instructions, and sequenced using paired end 2 × 300 bp version 3 chemistry on the MiSeq (Illumina) [33].

### MinION Library Preparation and Sequencing

For MinION sequencing, 47/58 isolates were successfully regrown as detailed above, DNA extraction carried out using FastDNA SPIN kit for Soil (MP Biomedicals) according to manufacturer's instructions, and quantified using the Nanodrop 1000 (ThermoFisher). See Supplementary Material for library preparation and sequencing methods.

### Sequence Alignment and Variant Calling

Sequence reads from both sequencing platforms were mapped to the FA1090 reference genome (RefSeq accession: NC_002946) using bwa mem (version 0.7.3a-r367), alignments sorted, duplicates removed with samtools and bcftools (version 1.3.1) [34], and variant calling performed using samtools mpileup. For phylogenetic analysis, site statistics were generated using samtools mpileup and sites filtered using the following criteria: mapping quality (MQ) above 30; site quality score (QUAL) above 30; at least four reads depth (DP) covering each site with at least two reads mapping to each strand; at least 75% of reads supporting site (DP4) (either the reference [REF] or a variant [ALT] base call); allelic frequency (AF1) of one. Sites that failed these criteria in any isolate were removed from analysis.

### Phylogenetic Reconstruction

Phylogenetic reconstruction was performed using RAxML (version 8.2.3) [35] with a GTR model of nucleotide substitution and a GAMMA model of rate heterogeneity; branch support values determined using 1000 bootstrap replicates. Phylogenetic placement of MinION sequence data was performed by generating site statistics as above, followed by tree estimation using RAxML. SNP distance, defined as the number of SNP differences between the MinION isolate at a particular depth and the corresponding isolate on the Illumina tree, was obtained using python package ete3.

### Sequencing Accuracy of MinION

MinION sequencing accuracy at varying depth of coverage was assessed by comparing MiSeq and MinION variant calling across 68 nucleotide positions that contribute to 37 well-characterized and putative non-plasmid resistance-associated markers (RAMs) for penicillin, ciprofloxacin, azithromycin, and tetracycline resistance (Table 1) [7, 15, 36].

**Table 1.** Resistance-Associated Markers

Gene	SNP Location	Amino Acid Change	Antibiotic
*gyrA*	620918	S91F	Cip
	620906	D95N/G	
*parC*	1210776	G85C	Cip
	1210779	D86N	
	1210782	S87R/I/N	
	1210785	S88P	
	1210794-5	E91K/G	
	1210870	R116L	
*rpsJ*	1807502	V57M	Tet
*pilQ*	103572	E666G	Tet, Pen
*penB* (porB1b)	1789055-7	G120K/D	Tet, Pen
	1789058-9	A121D/N	
*mtrR* promotor disruption (13-bp region)	1327741-1327753	A deletion	Tet, Azi, Pen
*mtrR* novel promotor	1327667	mtr120	Tet, Azi, Pen
m*trR*	1327931	G45D	Tet, Azi, Pen
	1327912	A39T	Azi, Pen
23sRNA (4 alleles)	1116542 1258645 1650221 1873373	C2611T	Azi
	1117097 1259197 1650773 1873925	A2059G	Azi
ponA	109929	L421P	Pen
Plasmid	…	Tet-M	Tet
Plasmid	…	blaTEM-1	Pen
	…	blaTEM-135	

Characterized resistance associated markers (RAMs) associated with ciprofloxacin (Cip), azithromycin (Azi), tetracycline (Tet), and penicillin (Pen) resistance in *Neisseria gonorrhoeae* (37 nonplasmid, and 3 plasmid). Gene name, single-nucleotide polymorphism (SNP) location (nucleotide position according to NC_002946 reference genome), and amino acid change or variation linked to resistance are detailed [7, 15, 36].

Isolates with MiSeq sequence depth of <30x, our gold standard, were excluded from analysis, as were MinION sequences not reaching a final depth of ≥40x, to account for differing sequencing durations and ensure unbiased retrospective comparison across different depths. Also excluded were two beta lactamase and tet-M plasmids, as their presence was expected to be determined near immediately. A “required sequencing depth,” defined as a suitable depth above which no significant improvement in accuracy of sequencing was achieved, was calculated as a surrogate for the shortest sequencing time providing accurate results.

### Accuracy of MinION Molecular Distances Between Isolates

Using the variable nucleotide positions used to construct the maximum-likelihood phylogenetic tree, SNP distances between pairs of whole-genome sequences were classified by estimated yearly evolutionary distance (YED), based on the literature described above. Thus, a distance of ≤5 SNP reflected a possible transmission pair, or isolates separated by one year of time. Subsequent YEDs were then defined by multiples of 5 SNP, therefore < 1 year, 1 to <2 years, 2 to <3 years, 3 to <4 years, 4 to <10 years, 10 to <50 years, and ≥50 years, corresponded to 0–4, 5–9, 10–14, 15–19, 20–49, 50–249, and ≥ 250 SNP distances, respectively [18].

MinION to MiSeq pairwise SNP distances were calculated by substituting in a MinION sequence for one of each pair, thus creating two MinION-MiSeq distances for each MiSeq-MiSeq distance and isolate pair. The discrepancy in SNP number between the two MinION-MiSeq distances for each MiSeq-MiSeq distance is defined as the SNP distance error. The YED categories were plotted against MinION-MiSeq SNP distance error, and ANOVA used to determine how the latter varied as YED increased.

### Post Hoc Quality Control Adjustment

A retrospective analysis to determine impact of relaxing variant calling MinION quality control (QC) parameters, for the 68 nucleotide positions, was performed using two alternative QC filters, which involved two levels of relaxation of the DP4 criteria which determines a reference (REF) or variant (ALT) base call. The impact was assessed as the number of base calls passing QC, and base calling accuracy compared to MiSeq sequencing. See Supplementary Material for detailed methodology.

### Ethics

All examined gonococcal isolates were collected as part of routine diagnostics (standard care) before being anonymized and submitted to GRASP. GRASP is a routine public health surveillance activity, and no specific consent is required from the patients. Patients are informed about GRASP at every participating site, using written notices. The UK Health Security Agency (UKHSA) has permission to handle data obtained by GRASP under section 251 of the UK National Health Service Act of 2006 (previously section 60 of the Health and Social Care Act of 2001), which was renewed annually by the ethics and confidentiality committee of the National Information Governance Board until 2013. Since then, the power of approval of public health surveillance activity has been granted directly to the UKHSA. Isolates were returned for culture and sequencing with only sample site and sex details used, therefore ethics approval was not needed.

## RESULTS

### *N. gonorrhoeae* Isolate Characteristics

Of 58 isolates grown and subsequently sequenced on MiSeq, 47 were successfully regrown, and sequenced on MinION. Two further isolates were excluded from analysis, one due to no MIC data being available, and another following analysis that suggested the MiSeq and MinION sequences came from different isolates, likely due to laboratory error. Thus MinION, MiSeq, and MIC data were initially available for 45 confirmed *N. gonorrhoeae* isolates (Table 2).

**Table 2.** *Neisseria gonorrhoeae* Isolate MIC Data

Isolate ID	Quinolone MIC, mg/L	Macrolide MIC, mg/L	Penicillin MIC, mg/L	Ceftriaxone MIC, mg/L	Cefixime MIC, mg/L
NG002	0.03	0.13	*0.25*	0.004	0.008
NG003	**16**	*0.5*	*1*	0.03	0.125
NG004	0.03	0.125	*0.5*	0.015	0.008
NG005	**>16**	0.25	**2**	0.03	0.125
NG006	0.03	0.06	*0.125*	0.004	0.008
NG007	**16**	**1**	*1*	0.06	0.06
NG008	**>16**	0.25	*1*	0.015	0.015
NG010	0.03	0.25	*1*	0.008	0.015
NG011	**8**	0.125	*0.25*	0.015	0.06
NG012	0.03	0.125	*0.25*	0.004	0.008
NG013	0.03	0.125	*0.125*	0.004	0.008
NG014	**16**	0.25	*0.5*	0.03	0.125
NG015	0.03	0.125	*0.125*	0.004	0.008
NG016	**16**	0.25	*1*	0.03	0.125
NG017	0.03	0.125	*0.25*	0.004	0.008
NG018	0.03	0.25	*0.25*	0.004	0.008
NG019	**>16**	0.25	*1*	0.008	0.008
NG020	0.03	0.125	*0.25*	0.004	0.008
NG022	**16**	*0.5*	*0.5*	0.03	0.06
NG023	**16**	0.25	*1*	0.03	0.125
NG024	0.03	0.06	*0.25*	0.008	0.06
NG026	0.03	0.25	*0.25*	0.008	0.008
NG027	0.03	0.125	*0.125*	0.008	0.008
NG028	0.03	0.03	*0.125*	0.004	0.008
NG029	0.03	0.125	*0.25*	0.004	0.008
NG031	0.03	0.25	*0.25*	≤0.002	0.004
NG032	0.03	0.03	*0.125*	≤0.002	≤0.002
NG033	0.03	0.125	*0.25*	≤0.002	0.008
NG035	0.03	0.25	*0.25*	0.004	0.008
NG037	**8**	0.125	*0.5*	0.015	0.06
NG038	**8**	0.125	*0.5*	0.015	0.06
NG039	0.03	0.06	*0.25*	≤0.002	0.008
NG040	0.03	0.06	*0.125*	0.004	0.008
NG043	0.03	0.125	*0.25*	0.004	0.008
NG044	0.03	0.03	≤0.060	0.004	0.015
NG045	0.03	0.125	*0.25*	0.004	0.008
NG047	0.03	0.125	*0.125*	≤0.002	0.008
NG048	0.03	0.03	≤0.060	≤0.002	≤0.002
NG049	0.03	**1**	**4**	0.06	0.06
NG050	0.03	**1**	**4**	0.06	0.06
NG051	0.03	*0.5*	**4**	0.06	0.06
NG052	0.03	0.03	*0.125*	≤0.002	0.008
NG054	0.03	0.125	*0.125*	0.004	0.008
NG056	0.03	0.25	*0.25*	0.004	0.008
NG057	0.03	0.125	**4**	0.004	0.008

Minimum inhibitory concentrations (MIC) for 45 *Neisseria gonorrhoeae* isolates. Quinolone MIC breakpoints defined as: susceptible ≤0.03 mg/L, intermediate >0.03 to ≤ 0.06 mg/L, and resistant >0.06 mg/L. Macrolide MIC breakpoints defined as: susceptible ≤0.25 mg/L, intermediate >0.25 to ≤ 0.5 mg/L, and resistant >0.5 mg/L. Penicillin MIC breakpoints defined as: susceptible ≤0.06 mg/L, intermediate >0.06 to ≤ 1 mg/L, and resistant >1 mg/L. Ceftriaxone and cefixime MIC breakpoints defined as: susceptible ≤0.125 mg/L and resistant >0.125. Bold, resistant; *italics*, intermediate; plain font, susceptible, according to European Committee on Antimicrobial Susceptibility Testing MIC breakpoints [32].

### MiSeq and MinION Sequencing

**Figure 1.**
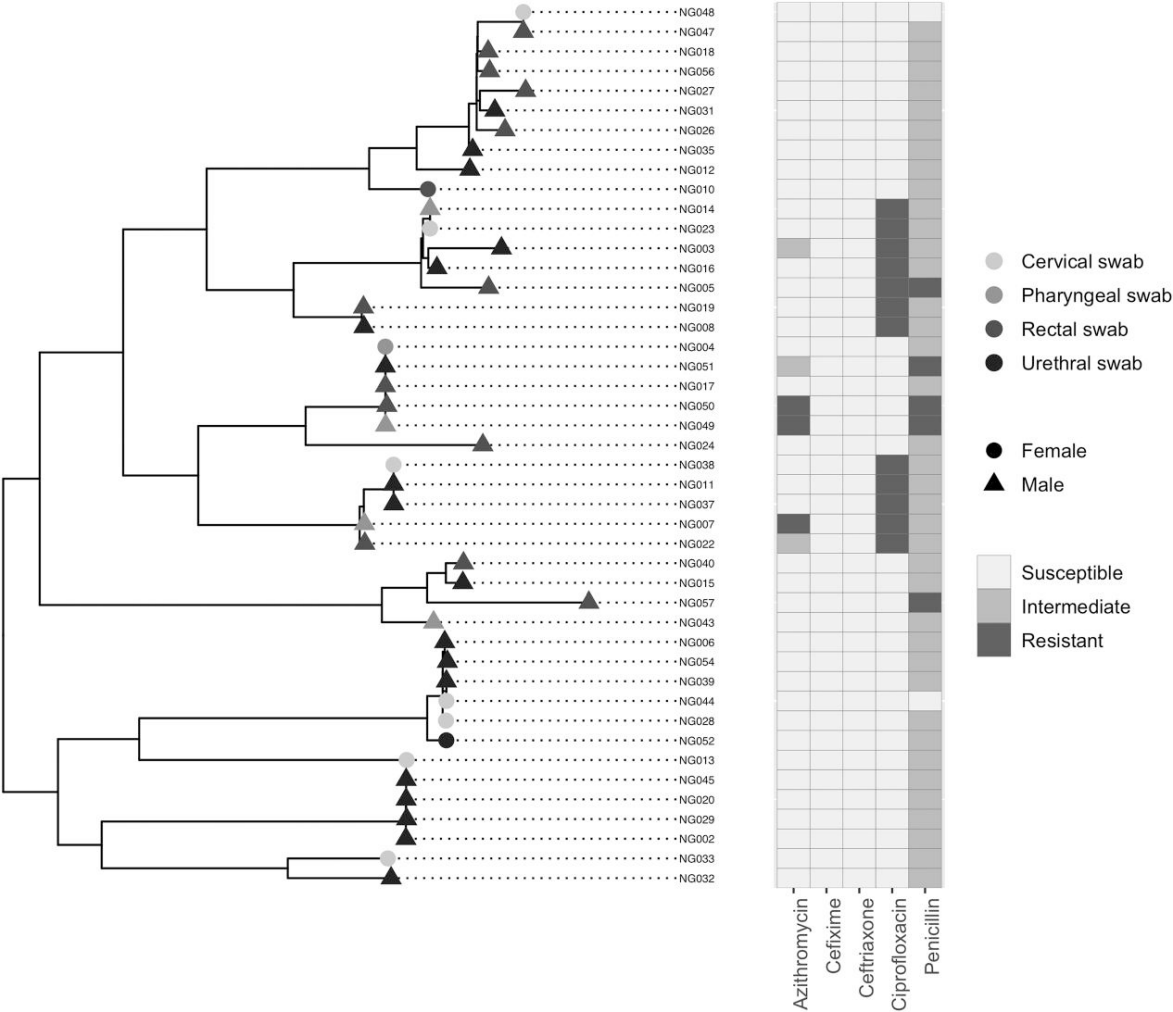
Phylogenetic tree of MiSeq whole-genome sequences. Phylogenetic and antimicrobial susceptibility/resistance comparisons of 45 gonococcal isolates. Terminal shapes represent sex of original sample source, and shading sample site. Phenotypic susceptibility profiles are shown as a heatmap as susceptible (white), intermediate (light grey), and resistant (dark grey).

Sequence characteristics for individual isolates, including median length of *N. gonorrhoeae* mapped reads and final depth of coverage achieved by MiSeq and MinION in all 45 isolates, are detailed in Supplementary Table 1. Phylogenetic reconstruction of MiSeq sequences enabled assessment of isolate diversity. Reference alignments were used to identify presence of known RAMs, and phenotype and sample sites were overlaid on the reconstruction (Figure 1). The relationship between sequencing depth and sequencing time is shown in Supplementary Figure 1.

### Genotypic Prediction of Resistance

The ability to identify known non-plasmid RAMs was assessed for 68 nucleotide positions in 22 isolate MiSeq and MinION sequence pairs, where MinION had reached a final depth of ≥40x. Retrospective analysis of these 22 isolate sequences at 10x, 30x, and 40x depth revealed the median number of 68 possible positions per isolate passing standard MinION QC was low at 8 (interquartile range [IQR], 5–11), 23 (IQR, 18–25), and 25 (IQR, 22–30) positions, respectively. Combining all nucleotide positions that passed MinION QC from all 22 isolate sequences, overall MiSeq variant call agreement was 100% (185/185; 95% confidence interval [CI], 98.0%–100.0%), 99.8% (502/503; 95% CI, 98.9%–99.9%), and 99.8% (564/565; 95% CI, 99.0%–100.0%) at 10x, 30x, and 40x MinION depth, respectively (Supplementary Table 2).

QC parameters for the 22 MinION isolate sequences at 10x depth were not passed for a nucleotide position within codon 91 of GyrA (genome nucleotide position 620918). However, at 30x and 40x depth, 10 and 12 of the 22 isolates, respectively, passed QC parameters, and of those 100% matched the sequence determined by MiSeq at the corresponding position, which included both REF and ALT calls. However, 4/22 isolates that failed QC for a nucleotide in codon 91 at 10x depth, and 13/22 at both 30x and 40x depth, all passed QC at a variant position within codon 95 of GyrA (genome nucleotide position 620906), with 100% agreement to the MiSeq data (Supplementary Table 2C). In this position, all calls passing QC accurately were REF calls only.

In addition, there were insufficient high-quality variant calls covering the 13-bp repeat region within the *mtrR* promotor region to identify a single base deletion in the 5-A repeat region, present in 5/22 isolates, based on the MiSeq variant calling results.

### Identification of Sequence Clusters

A maximum-likelihood phylogenetic tree was constructed, using 7238 variable nucleotide positions present in consensus MiSeq sequences in all 45 isolates that met sequencing QC parameters (Figure 1). Accuracy of placement of individual MinION consensus sequences on to the MiSeq tree was assessed by measuring the MiSeq-MinION pairwise SNP distance of the same isolates.

**Figure 2.**
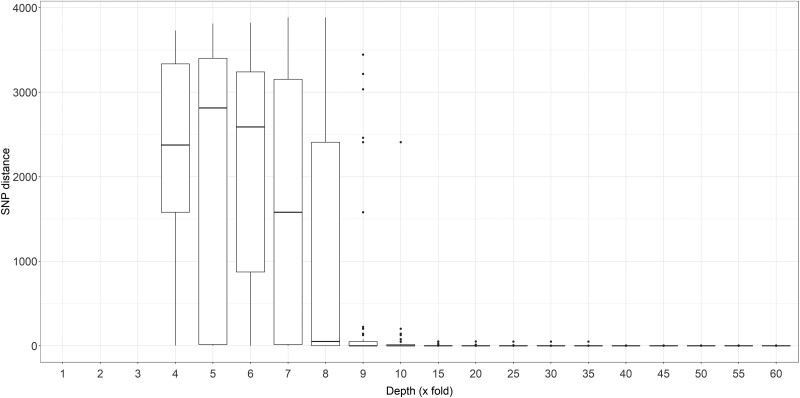
Single-nucleotide polymorphism (SNP) distances between isolate MinION and MiSeq sequences. Box and whisker plot representing median and interquartile range of SNP distances between MinION and MiSeq sequences of the same isolate, at increasing MinION sequencing depth of coverage.

**Figure 3.**
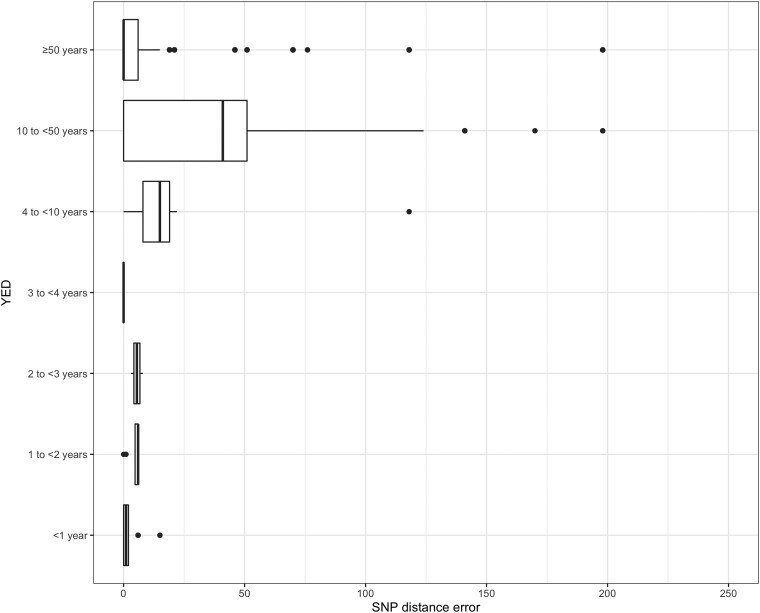
Phylogenetic accuracy of MinION 10x sequencing depth, measured in yearly evolutionary distances (YEDs). Pairwise single-nucleotide polymorphism (SNP) distances between MiSeq-MiSeq whole-genome sequences grouped by YED (assumption, 1 YED = 5 SNPs). Two corresponding MiSeq-MinION SNP distances (created by substituting each of the isolates in the MiSeq-MiSeq pairs for its MinION sequence) were compared to the MiSeq-MiSeq SNP distances (of the same isolates). The difference seen by MinION sequence introduction, known as the SNP distance error, is represented as median and interquartile range by the box and whisker plot. Median YED count for each category: ≥ 50 years, 1848; 10 to <50 years, 66; 4 to <10 years, 18; 3 to <4 years, 2; 2 to <3 years, 2; 1 to <2 years, 8; and < 1 year, 36.

Figure 2 shows the SNP distances over increasing average MinION sequencing depth. SNP distances reduced considerably up to a sequencing depth of 15x (median SNP distances were depths 9x, 0.70; 10x, 0.20; and 15x, 0.08). Based on this and the increased sequencing time needed to achieve 15x depth of coverage, we selected 10x as the required sequencing depth. A depth of 10x corresponded to a median of 29 minutes (IQR, 25–39 minutes) sequencing time (Supplementary Figure 1). For the 45 isolates, SNP distances between all MiSeq pairs (45 × 44; n = 1980), not including self-comparison, ranged from 0 to 4053 SNP. Following categorization of SNP distances between pairs of whole-genome sequences into YEDs, most pairs were separated by more than 50 years of molecular distance, and 18 pairs were closely related within 1 YED. For each MiSeq-MiSeq pair, two MinION-MiSeq SNP distances were calculated (Figure 3).

Despite median pairwise SNP distance error between MinION-MiSeq and MiSeq-MiSeq sequences being similar across all YED categories: 1 (IQR, 0–2), 6 (IQR, 5–6), 6 (IQR, 4–7), 0 (IQR, 0–0), 15 (IQR, 8–19), 41 (IQR, 0–51), and 0 (IQR, 0–6) for <1 year, 1 to <2 years, 2 to <3 years, 3 to <4 years, 4 to <10 years, 10 to <50 years, and ≥50 years, respectively (*P* = .913; Figure 3). MinION-MiSeq SNP distance errors of >50 were seen only in YED categories 10 to <50 and ≥50. Within YED category of <1 year, there were 6 isolate pairs with an SNP distance error of 6, and 2 with an SNP distance error of 15.

### Post Hoc Quality Control Adjustment

A post hoc exploration into low MinION QC rates in RAMs suggested the stringency of the QC parameters used, possibly played a part in several variant calls being missed. The largest proportion of all variant calls failing QC did so due to insufficient MQ and DP4 values, particularly due to so-called strand bias. As expected, relaxing QC parameters increased pass rates (Supplementary Table 2). See Supplementary Material for detailed results.

## DISCUSSION

Our study suggests that nanopore sequencing can accurately perform flexible and timely identification of closely related gonococcal strains, offering potential to detect transmission networks. Concerns around drug-resistant gonorrhea, particularly to extended-spectrum cephalosporins [37], suggest evaluations of established and novel surveillance approaches in or near clinic are warranted. The low cost and portability of the MinION platform facilitates rapid sequencing in remote areas, with the development of methods for sequencing directly from samples enabling the use of sequencing information in relative real-time with consumable costs likely to become affordable for routine use [38].

As expected, nanopore sequencing accuracy as compared with MiSeq increased progressively with increasing depth. However, as little as 30 minutes of nanopore sequencing, equivalent to 10x average sequencing depth, identified closely related gonococcal strains determined by MiSeq sequencing as our gold standard comparison, where final read depths were >30x. We chose a conservative estimate of five SNP to represent an evolutionary distance of one year between strains, with previous studies suggesting such a distance may also be applicable to determining likely transmission pairs [18, 19].

We were unable to directly compare MinION and MiSeq phylogenetic tree topologies, as too few common SNP among the MinION sequences passed QC. Instead, we calculated SNP distances between every possible pair of MiSeq sequences, categorized them into YEDs, and compared them to MinION-MiSeq SNP distances for the same pairs (ie, two distances per pair). MinION sequencing at 10x depth accurately measured 28 of the 36 MinION-MiSeq distances, representing the 18 pairs of isolates that fell within a molecular distance of less than one year, with a further two distances still falling within a less conservative measure for a year of molecular clock [18]. None of the pairs categorized by MiSeq-MiSeq as greater than one year were incorrectly assigned to the <1 YED category by the MinION. Although, statistically there was no difference between SNP distance errors across YED categories, more outliers were seen with increasing yearly distance, a reflection of the increasing variant number and low read depth used.

This study was not designed to demonstrate accuracy of predicting AMR phenotype for multiple classes of antibiotics, due to the complexity of having multiple determinants for some antibiotic classes [39]. We did, however, evaluate the ability to correctly call specific RAMs associated with AMR, which were comparable at 10x, 30x, and 40x MinION depth. Previous work has demonstrated a WGS-based MIC prediction model, which successfully predicted MIC values with between 90.4% and 98.2% accuracy within ­_­_±1 doubling dilution, dependent on antibiotic class, and may be adaptable to lower sequencing depths [15, 17]. The AMR RAMs chosen for this analysis were not an exhaustive list and were selected for being either well characterized or putative within the prevailing literature at the time. Thus, recent resistance markers such as the mosaic *penA* carried in the FC428 clone associated with ceftriaxone resistance was not included, being identified in 2015. Furthermore, up until then very few isolates had been demonstrating ceftriaxone or cefixime resistance phenotypes [40].

Despite the accuracy of rapid nanopore sequencing for specific RAMs being high overall, the large number of positions that did not pass standard QC parameters would not allow for this version of the technology to be used for AMR diagnostic purposes; however, this is likely to have changed with improved versions of the platform and flow cell technology. Some RAMs, such as the deletion of a single adenine in the *mtrR* promotor region, were not detected due to poor QC pass rates and the challenges for MinION handling of homopolymers. However, more recent versions of the platform have addressed this problem to some degree [41].

The high QC failure rate prompted post hoc reanalysis with less stringent QC criteria for the RAMs. These increased the number of RAMs that could be evaluated without losing accuracy, except in the case of the least stringent QC parameters, filter 2. However, QC rates were still high, exemplified by the nucleotide positions corresponding to the S91 GyrA codon position, a high-confidence marker for ciprofloxacin susceptibility, where at 10x depth the number of isolates passing QC at this position increased from 0 to 7, with 100% accuracy.

This study used gonococcal isolates from culture and did not perform bioinformatic analysis in real time. Future approaches will likely need to use techniques such as DNA capture to facilitate sequencing directly from samples [24], or more general metagenomic approaches [28], and remove the need for culture, to reduce overall time taken. Automated bioinformatic pipelines will also enable data interrogation in real time, for example, the ARTIC network RAMPART (Read Assignment, Mapping, and Phylogenetic Analysis in Real Time) software, a bioinformatics protocol for the analysis of nanopore sequences [42].

Nanopore technology is undergoing continuous evolution, upgrading the performance of its sequencing technology via improved accuracy in base-calling algorithms, increased throughput and capture, and better raw read accuracy [41, 43]. Additionally, newer rapid library preparation methods may also contribute to reducing the time taken from sample to sequence. The Flongle flow cell is smaller and cheaper than the conventional MinION flow cells, and adaptable to lower sample number applications such as this. The more recent R10 nanopore further improves the accuracy for detecting homopolymers, and the transition from Hidden Markov Models-based approach to a deep learning approach have improved the read accuracy dramatically [41]. Research is ongoing into sequencing directly from clinical samples, with Street et al reporting successful extraction and sequencing of *N. gonorrhoeae* DNA directly from urine samples, achieving a coverage of ≥92.8% across the whole genome in 10 patient samples, and ≥93.8% at ≥10× sequencing depth in 7 [24]. The combination of these improvements suggests that MinION offers promise as a tool to widen rapid *N. gonorrhoeae* surveillance for monitoring AMR spread.

In conclusion, MinION sequencing was able to accurately determine closely related gonococcal strains, related within a molecular distance of 1 year, with as little as 10× average depth of coverage, demonstrating the potential for its application as a real-time practical surveillance tool.

## Supplementary Data

Supplementary materials are available at *The Journal of Infectious Diseases* online. Consisting of data provided by the authors to benefit the reader, the posted materials are not copyedited and are the sole responsibility of the authors, so questions or comments should be addressed to the corresponding author.

## Supplementary Material

jiad170_Supplementary_Data

## Notes

***Acknowledgments.*** We thank the staff and patients of The Courtyard Clinic, St George's Healthcare NHS Trust. We also thank the clinical and laboratory staff at the UK Health Security Agency who contributed to the Gonococcal Resistance to Antimicrobials Surveillance Programme for returning the isolates used in this study.

***Financial support*.** This work was supported by the UK Clinical Research Collaboration (Medical Research Council) and Translation Infection Research Initiative Consortium (grant number G0901608).

***Potential conflicts of interest*.** S. T. S. has previously received consultancy fees from Phillips, Roche, and Abbott Diagnostics. All other authors report no potential conflicts.

***Data availability*.** Sequence data has been submitted to the ENA database with accession number PRJEB50649.
