# Supplementary material for: Time Required for Nanopore Whole-Genome Sequencing of Neisseria gonorrhoeae for Identification of Phylogenetic Relationships
Source: J Infect Dis. 2023 May 22;228(9):1179–88. doi: 10.1093/infdis/jiad170 (PMC10629711; doi:10.1093/infdis/jiad170)
Supplement: jiad170_Supplementary_Data [file jiad170_supplementary_data.zip › Supplementary_Table_1v2.docx]

**Supplementary Table 1.** **Summary of MinION and MiSeq sequencing outputs**

Read count and depth of coverage of MinION and MiSeq sequencing, with median read length for MinION only. Sequencing depth was calculated based on reads mapped against the FA1090 reference genome (accession: NC_002946).

| **Isolate** | **MiSeq** | | **MinION** | |  |
| --- | --- | --- | --- | --- | --- |
|  | Read count | Depth | Read count | Depth | Median read length |
| NG002 | 286726 | 59 | 37791 | 95 | 5228 |
| NG003 | 374461 | 80 | 32921 | 89 | 5906 |
| NG004 | 359432 | 79 | 32297 | 80 | 4724 |
| NG005 | 398056 | 84 | 16470 | 42 | 5268 |
| NG006 | 329834 | 75 | 66837 | 80 | 2013 |
| NG007 | 382478 | 79 | 37944 | 82 | 4477 |
| NG008 | 144410 | 26 | 58890 | 76 | 1957 |
| NG010 | 181701 | 41 | 28766 | 50 | 3489 |
| NG011 | 254192 | 55 | 21676 | 40 | 4016 |
| NG012 | 235162 | 54 | 29130 | 39 | 2491 |
| NG013 | 125864 | 22 | 37164 | 75 | 4311 |
| NG014 | 249449 | 54 | 36783 | 57 | 3096 |
| NG015 | 223409 | 46 | 19411 | 47 | 5598 |
| NG016 | 209404 | 45 | 20056 | 41 | 3802 |
| NG017 | 142738 | 30 | 21572 | 39 | 3882 |
| NG018 | 186799 | 42 | 40262 | 90 | 4891 |
| NG019 | 287033 | 63 | 32981 | 68 | 4416 |
| NG020 | 279821 | 60 | 84971 | 105 | 1813 |
| NG022 | 251790 | 53 | 39907 | 65 | 3409 |
| NG023 | 276267 | 62 | 57729 | 80 | 2488 |
| NG024 | 354985 | 75 | 26719 | 59 | 4767 |
| NG026 | 240317 | 54 | 19265 | 43 | 4865 |
| NG027 | 283617 | 61 | 26274 | 54 | 4487 |
| NG028 | 204630 | 42 | 36560 | 51 | 2559 |
| NG029 | 121248 | 21 | 13602 | 30 | 5154 |
| NG031 | 273662 | 63 | 13538 | 29 | 4489 |
| NG032 | 326198 | 68 | 5117 | 11 | 4299 |
| NG033 | 164870 | 37 | 21286 | 24 | 1883 |
| NG035 | 185770 | 38 | 18782 | 26 | 2131 |
| NG037 | 274239 | 61 | 17889 | 26 | 1658 |
| NG038 | 197600 | 41 | 27064 | 34 | 1755 |
| NG039 | 241124 | 54 | 15144 | 19 | 1774 |
| NG040 | 237790 | 51 | 8086 | 19 | 5672 |
| NG043 | 169118 | 28 | 80153 | 67 | 1355 |
| NG044 | 220790 | 45 | 27641 | 32 | 1636 |
| NG045 | 190776 | 43 | 26785 | 36 | 2043 |
| NG047 | 173802 | 39 | 32648 | 34 | 1412 |
| NG048 | 253662 | 56 | 25489 | 31 | 1576 |
| NG049 | 88812 | 17 | 39523 | 40 | 1499 |
| NG050 | 34598 | 6 | 45642 | 46 | 1188 |
| NG051 | 382287 | 83 | 24154 | 32 | 2067 |
| NG052 | 510395 | 111 | 49371 | 42 | 1500 |
| NG054 | 275550 | 58 | 47080 | 40 | 1427 |
| NG056 | 314907 | 71 | 13558 | 32 | 5172 |
| NG057 | 169721 | 31 | 15576 | 32 | 4892 |
