# Supplementary material for: Time Required for Nanopore Whole-Genome Sequencing of Neisseria gonorrhoeae for Identification of Phylogenetic Relationships
Source: J Infect Dis. 2023 May 22;228(9):1179–88. doi: 10.1093/infdis/jiad170 (PMC10629711; doi:10.1093/infdis/jiad170)
Supplement: jiad170_Supplementary_Data [file jiad170_supplementary_data.zip › Supplementary_Table_2.docx]

**Supplementary Table 2. Number and accuracy of MinION variant calls passing standard and alternative QC parameters**

Total number of the 68 nucleotide positions representing 37 non-plasmid RAMs across 22 MinION isolates which: **A**. passed standard, filter 1, and filter 2 QC parameters **B**. passed standard, filter 1, and filter 2 QC parameters, and were accurate when compared to the MiSeq sequences within the same isolate, at the same position **C**. Total number of positions: 620906, and 620918 corresponding to GyrA D95 and S91 respectively, that passed standard, filter 1, and filter 2 QC parameters, and were accurate when compared to the MiSeq sequences within the same isolate, at the same position.

**A.**

| **Passed QC** | Standard QC | Filter1 | Filter2 |
| --- | --- | --- | --- |
| 10x | 13.5% | 28.7% | 52.8% |
|  | 185/1371 | 393/1372 | 724/1371 |
| 30x | 36.5% | 47.9% | 70.2% |
|  | 503/1378 | 660/1378 | 967/1378 |
| 40x | 40.9% | 51.3% | 72.7% |
|  | 565/1382 | 709/1382 | 1005/1382 |

**B**.

| **Passed QC + accuracy** | Standard QC | Filter1 | Filter2 |
| --- | --- | --- | --- |
| 10x | 100% | 100% | 99.2% |
|  | 185/185 | 393/393 | 718/724 |
| 30x | 99.8% | 99.8% | 98.8% |
|  | 502/503 | 659/660 | 955/967 |
| 40x | 99.8% | 99.7% | 98.8% |
|  | 564/565 | 707/709 | 993/1005 |

**C.**

Position: 620906 (GyrA D95)

| **Passed QC + accuracy** | Standard QC | Filter1 | Filter2 |
| --- | --- | --- | --- |
| 10x | 100% | 100% | 86.7% |
|  | 4/4 | 9/9 | 13/15 |
| 30x | 100% | 100% | 81.3% |
|  | 13/13 | 13/13 | 13/16 |
| 40x | 100% | 100% | 81.3% |
|  | 13/13 | 13/13 | 13/16 |

Position: 620918 (GyrA S91)

| **Passed QC + accuracy** | Standard QC | Filter1 | Filter2 |
| --- | --- | --- | --- |
| 10x | 0 | 100% | 100% |
|  | 0/0 | 7/7 | 13/13 |
| 30x | 100% | 100% | 100% |
|  | 10/10 | 14/14 | 16/16 |
| 40x | 100% | 100% | 100% |
|  | 12/12 | 14/14 | 17/17 |
