# Supplementary figures and images for: Time Required for Nanopore Whole-Genome Sequencing of Neisseria gonorrhoeae for Identification of Phylogenetic Relationships
Source: J Infect Dis. 2023 May 22;228(9):1179–88. doi: 10.1093/infdis/jiad170 (PMC10629711; doi:10.1093/infdis/jiad170)

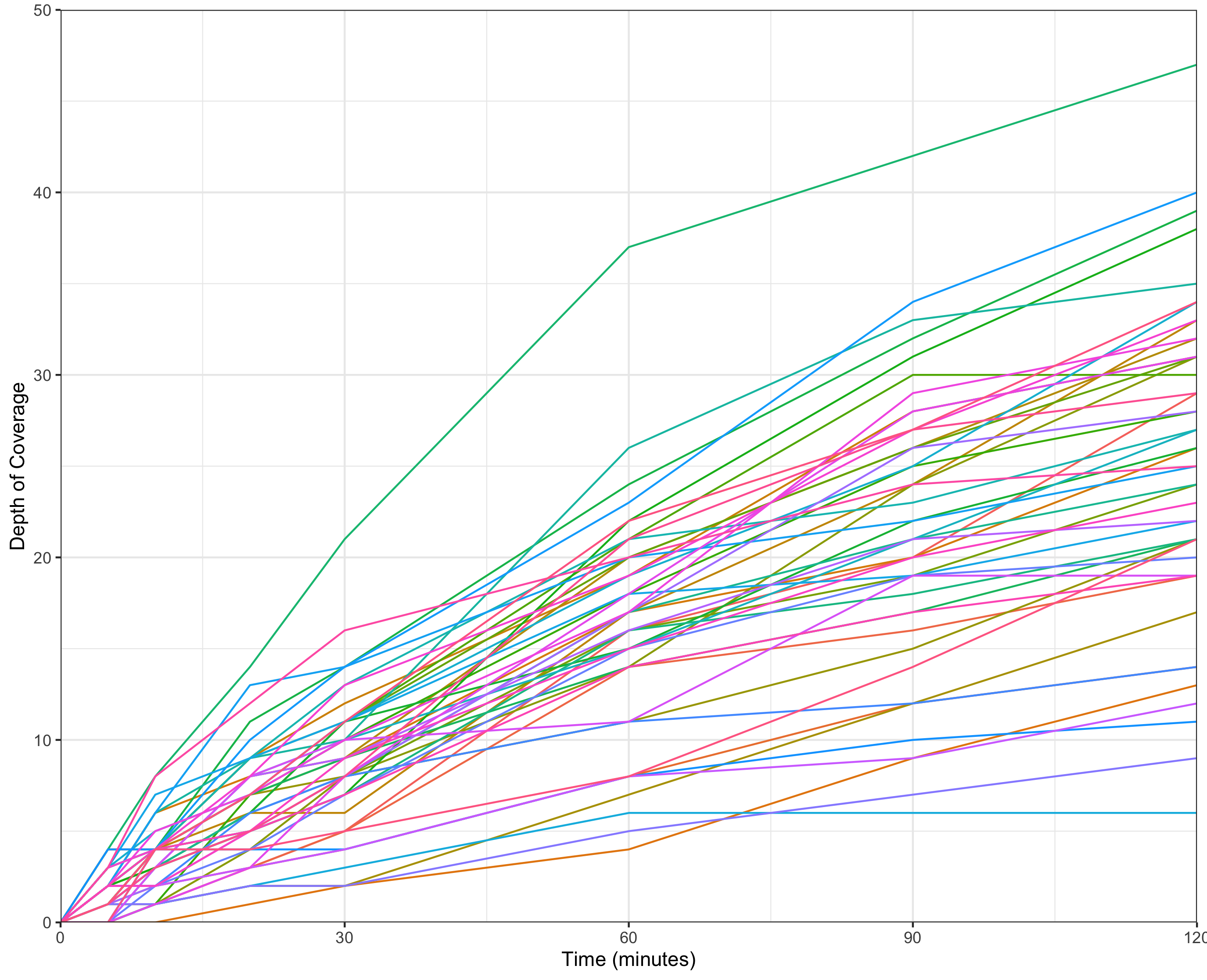

Supplement: jiad170_Supplementary_Data [file jiad170_supplementary_data.zip › Supplementary_figure_1 (1).tiff]
